# Characterization of the complete chloroplast genome of *Polygonatum sibiricum* (Liliaceae), a well-known herb to China

**DOI:** 10.1080/23802359.2019.1704193

**Published:** 2020-01-14

**Authors:** Jiaoyi Pan, Weijia Lu, Shishi Chen, Tianyi Cao, Linfeng Chi, Fule He

**Affiliations:** a Zhejiang University of Chinese Medicine, Hangzhou, Zhejiang, China;; b Guangzhou University of Chinese Medicine, Guangzhou, Guangdong, China;; c Department of Orthopaedics, Zhejiang Integrated Traditional Chinese and Western Medicine Hospital, Hangzhou, Zhejiang, China

**Keywords:** *Polygonatum sibiricum*, Liliaceae, chloroplast genome, phylogenetic relationship

## Abstract

*Polygonatum sibiricum* is a famous and well-known TCH (Traditional Chinese Herb) in China. In this paper, the complete chloroplast genome of *P. sibiricum* was studied and illustrated to add more genetic information and data. The chloroplast genome is 152,960 bp in length and a typical quadripartite structure, which exhibits a large single-copy region (LSC) of 81,471 bp, a small single-copy region (SSC) of 18,485 bp and a pair of inverted-repeat regions (IRs) of 26,502 bp in each. The overall nucleotide composition of chloroplast genome is: 30.7% A, 31.4% T, 19.3% C, 18.6% G and the total GC content 37.9%. A total of 136 genes were annotated that included 90 protein-coding genes (PCGs), 38 transfer RNA (tRNAs) and 8 ribosome RNA (rRNAs). The phylogenetic ML tree shown that *P. sibiricum* is closely related to *P. cyrtonema* on genetic position relationship by the Maximum-Likelihood (ML) method.

*Polygonatum sibiricum* is a well-known traditional Chinese herb, which had been widely applied for hundreds of years to treat many diseases in China, Korea, Japan, and other East Asian countries (Zhao and Li 2015). *Polygonatum sibiricum* has become endangered due to the uncontrolled excavation of natural resources in the world (Virk et al. 2016). The steroidal saponins, flavones, alkaloids, lignins, amino acids carbohydrates and so on is in *P. sibiricum*, which has potential anti-tumor and anti-viral proteins for possible medical application and large-scale pharmaceutical production (Zhao and Li 2015). At present, it is little known about the genome information of *P. sibiricum* that can provide more data available to study this species. In this paper, we had been finished the chloroplast genome of *P. sibiricum*, which can be useful for study the medicinal valuable and research the drug development of the family Liliaceae in future.

Using the Plant Tissues Genomic DNA Extraction Kit (TIANGEN, BJ and CN) method, total genomic DNA was isolated from the fresh stem of *P. sibiricum* and collected from herb market near Zhejiang Chinese Medical University that located at Hangzhou, Zhejiang, China (30.09N, 119.89E). The chloroplast genome DNA was stored in Zhejiang Chinese Medical University (No. SCMC-ZJU-TCM-05). And, it was purified and sequenced by the sequencer that the collected raw sequences were quality controlled and removed by the FastQC (Andrews 2015). The chloroplast genome of *P. sibiricum* was assembled and annotated by the MitoZ (Meng et al. 2019). The chloroplast genome map was generated by the OrganellarGenomeDRAW (Lohse et al. 2013).

The chloroplast genome of *P. sibiricum* (KT6956052) is a 152,960 base pairs (bp) long and has a typical quadripartite structure. It consists of a large single-copy region (LSC of 81,471 bp), a small single-copy region (SSC of 18,485 bp) and a pair of inverted repeat regions (IRs of 26,502 bp in each). The overall nucleotide composition of chloroplast genome is: 30.7% of A, 31.4% of T, 19.3% of C, 18.6% of G and the total GC content of 37.9%. The chloroplast genome of *P. sibiricum* contains 136 genes, which includes 90 protein-coding genes (PCG), 38 transfer RNA genes (tRNAs) and 8 ribosomal RNA genes (rRNAs). Twenty-one genes were found duplicated in each IR region, which includes 9 PCGs species (*rpl2, rpl23, ycf2, ycf15, ndhB, rps7, rps12, ycf68 a*nd *ycf1*), 8 tRNAs species (*trnH-GUG, trnI-CAU, trnL-CAA, trnV-GAC, trnI-GAU, trnA-UGC, trnR-ACG* and *trnN-GUU*) and 4 rRNAs species (*rRNA16, rRNA23, rRNA4.5* and *rRNA5*).

To confirm the phylogenetic position of *P. sibiricum*, the complete chloroplast sequences of 15 the family Liliaceae species were aligned using the MEGA X software (Kumar et al. 2018) and the maximum-likelihood (ML) method was constructed the phylogenetic tree. ML analysis of the phylogenetic tree was performed using with GTR + G + I model and all of the nodes were inferred with strong support by 2000 bootstrap values replicate for each node. The phylogenetic tree was represented using the MEGA and edited using the Evolview online (www.evolgenius.info/evolview) (Subramanian et al. 2019). The phylogenetic ML tree showed that *P. sibiricum* is closely related to *Polygonatum cyrtonema* (NC_028429.1) on genetic position relationship (Figure 1). This result offers is important to study the medicinal valuable and research the biosynthesis, metabolism, and regulation of this species.

**Figure 1.**
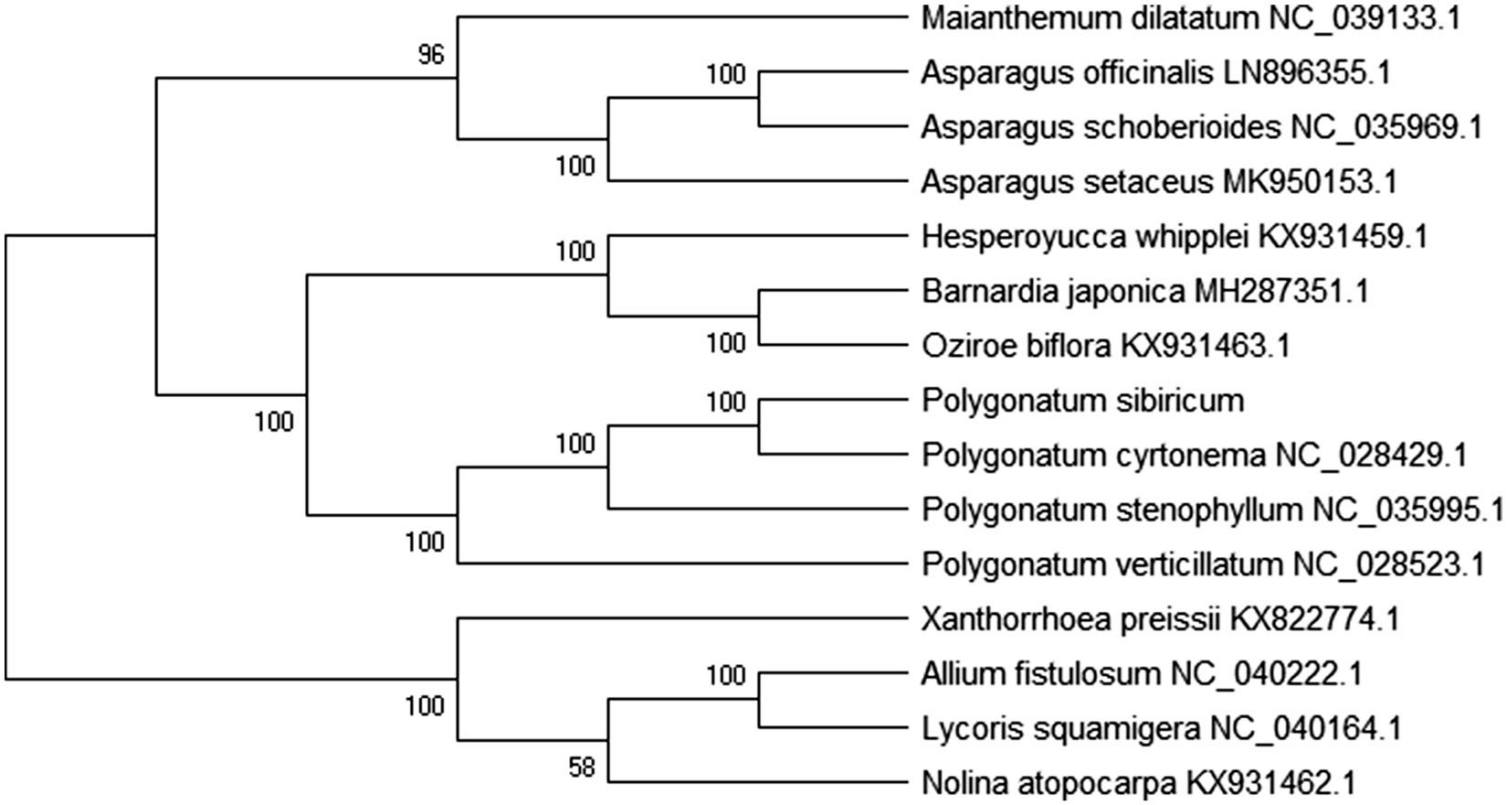
The phylogenetic maximum-likelihood tree of *Polygonatum sibiricum* based on 15 the family Liliaceae species chloroplast genomes. All the nodes are the bootstrap values from 2000 replicates.
